# Better nanoscience through open, collaborative, and critical discussions

**DOI:** 10.1039/d3mh01781h

**Published:** 2024-04-05

**Authors:** Nathanne Cristina Vilela Rost, Maha Said, Mustafa Gharib, Raphaël Lévy, Federico Boem

**Affiliations:** a Université Sorbonne Paris Nord and Université Paris Cité, INSERM, LVTS F-75018 Paris France; b University of Twente, Philosophy Section, Drienerlolaan 5 7522 NB Enschede The Netherlands f.boem@utwente.nl

## Abstract

We aim to foster a discussion of science correction and of how individual researchers can improve the quality and control of scientific production. This is crucial because although the maintenance of rigorous standards and the scrupulous control of research findings and methods are sometimes taken for granted, in practice, we are routinely confronted with articles that contain errors.

## Abstract 2

Material science publications are the outcome of research, but they can contain errors. We advocate for post publication peer review as a way to collectively improve self-correction of science.

## Graphical abstract

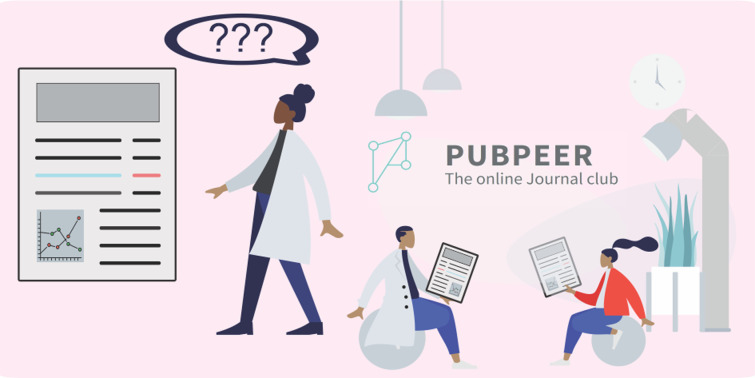

## Errors and self-correction of science

The issue of lack of reproducibility and limited translation of research findings is a recurrent concern in nanoscience.^1–3^ Since the development of the so-called ‘modern science’ in the 17th century, the scientific enterprise has been founded on the public research dimension.^4^ This means that scientific work is not conducted privately but actively involves communities, *i.e.*, groups of people animated by similar aims and shared methods, constituting both the ‘government’ of the scientific enterprise and also its controlling body. Moreover, the collegial and open character of scientific research hinges on the idea that any scientific statement, before it can be recognised and accepted as such, must pass critical scrutiny that is carried out by the scientific community itself. The scientific culture in which researchers are trained and immersed should, ideally, foster the creation of a critical mindset characterised by the need to evaluate research claims based on the internal consistency of experimental hypotheses, models, theories and on methodological rigour. Therefore, the scientific community is not only responsible for establishing the soundness of its studies but also for checking for any flaws, which may have escaped earlier levels of control, and correcting them. This concept is what the sociologist of science, Robert K. Merton, called ‘organized skepticism’^5^ by which he emphasises that although it is necessary for theoretical and experimental production to meet methodological criteria, it is not sufficient to establish a ‘scientific fact’.

One of the levels where critical scrutiny takes place is peer review, but that process is often relatively superficial, and it certainly does not involve independently reproducing experimental studies. As a result, errors (both involuntary and, in some cases, intentional) do pass the filter of peer review (see ref. 6–9). The few published studies of the peer review process itself show its limitations. For example, research carried out in the British Medical Journal on 607 trained peer reviewers revealed that they detected less than half of the errors regardless of the type of training they had received.^10^ Although science is supposed to be self-correcting, once peer-reviewed articles are published, scientists have little incentive to correct or to critically analyse them.^11–13^ Indeed, in the contemporary scientific landscape, the incentives that drive researchers play a key role in shaping the trajectory of scientific enquiry and publication practices. These incentives, due to the interests involved, could favour the pursuit of discoveries presented as revolutionary and attention-grabbing over meticulous but less conspicuous work. However, different contexts with different incentive and disincentive mechanisms may indicate that certain dynamics and patterns are not always generalisable and should always be analysed specifically. On the other hand, a research landscape that is still too marked by structural inequalities and working conditions that are not always adequate can exacerbate these situations.^14,15^

One of the main methods of correcting science concerns the possibility of withdrawing a problematic article. However, a recent analysis of this phenomenon shows that, at least as far as chemistry and material sciences are concerned, this is largely restricted to cases of scientific fraud such as plagiarism or data manipulation.^16^ Moreover, researchers who attempt to publish a negative study or even challenge the state-of-the-art,^17–20^ or to engage critically with the literature, generally find it to be a complex and protracted process (see ref. 21 and 22). Various factors, such as: questionable research practice (QRP), personal biases, limited resources, and societal pressures, including fear of retaliation and the limited recognition of such activities in the evaluation of researchers, may influence the weaknesses of self-correction mechanisms (Table 1).

**Table 1:** Examples of factors hindering self-correction

QRPs and factors hindering self-correction	Ref.
Metrics for evaluation and hiring	26
Unextensive (insufficient) knowledge of the literature leading to poor quality of research	27
Academic prestige/authority (Academic leadership)	26
Competition/no will for enemies	28
The fear for the public image of science (Trust in science)	29
Malpractice/sloppiness	30
Pressure to publish	30
Lack of transparency	31–33
P-hacking	34

Thus, the overwhelming majority of published results are “positive”, either because what was hypothesised from the start of the study was indeed confirmed or, in many cases, because of ‘Hypothesising After the Results are Known’ also known as HARKing.^23^ In contrast, “negative” results, *i.e.* results that refute the suggested hypothesis or that do not have the same tendencies as other results in the study,^23^ are more often ignored, or their importance downplayed. Recognizing the pervasive challenge of publication bias, where positive results often overshadow negative findings, the scientific community grapples with the ‘file drawer problem’—a phenomenon wherein negative results are frequently ignored or downplayed,^24^ contributing to an incomplete portrayal of research outcomes. This affects the potential of machine learning and artificial intelligence to generate predictions based on automated analysis of the literature, since they can only provide useful insights if the scientific record is not marred by biases and errors (alternatively, those biases and errors need to be documented and taken into account). This is illustrated by a recent study by Beker *et al.* who showed that based on a set of over 10 000 selected publications, machine learning models fail to provide useful predictions of optimum reaction conditions for heterocyclic Suzuki–Miyaura coupling.^25^

Uncorrected errors in the literature can generate ‘bubbles’. Möhwald and Weiss^35^ eloquently evoked this possibility in a 2015 ACS Nano editorial entitled “*Is Nano a Bubble?*”. For example, the widely discussed perspective article by Wang and colleagues^36^ entitled “*Will Any Crap We Put into Graphene Increase Its Electrocatalytic Effect?*” describes an example of a scientific bubble (without using the word) where thousands of articles are published on the basis of constantly renewed promises leading to little progress in scientific understanding and no improvement in technological performance. A bubble may be created not so much because of genuine scientific interest but rather because it impacts the earnings of investors^37–39^ or because its novelty would foster specific ways of doing research.^40^ Whilst the tone of Wang and colleagues (and indeed that one of Weiss and Möhwald) is refreshingly light, the consequences of those bubbles can be serious, such as raising unrealistic hopes among patients or resulting in wasted research funding based on misconceptions. These examples illustrate that distorted or exaggerated scientific claims are not uncommon in the scientific literature. Knowing how to distinguish and assess when a certain use of language may generate distortions or false expectations becomes crucial to prevent the formation and growth of ‘bubbles’.

## Post-publication peer review

As a step towards achieving this aim, we argue here in favour of integrating an additional level of control, *i.e.* post-publication peer review, as a routine part of the work we do as scientists. As the expression indicates, post-publication peer review consists of providing feedback and comments on an article after it has been published.^41–43^ This ‘second’ review process takes place in a public online forum and could be carried out by any researcher willing to contribute, anonymously or not. Post-publication peer review broadens the community of peers who can express themselves on the quality of an article, enabling the identification and discussion of problems and offering a potential mechanism for making the certification of a result more robust. Thus, it provides a digital space where the scientific community can gather to engage in discussions openly and transparently about methods, results and interpretations. This reflects, at least ideally, the famous image (after the so-called Scientific Revolution) of the scientific community, meaning scholars metaphorically belonging to a common research network which shares standards, methods, and discusses them (see ref. 4 and 17). By relying on a broader, collective revision system, post-publication peer review could provide a helpful tool for evaluating a scientific hypothesis in a more critical, timely and lucid way.^41,43^

Critical analysis of articles is everyday work in research laboratories, *e.g.*, to evaluate methodologies, establish new protocols and survey the state-of-the-art. When researchers read an article to formulate ideas for their projects, they may often observe flaws, unclear or incomplete methods, or a lack of data explanation. Usually, those observations are stored as private notes, which are at best shared within a single research group or with a few colleagues.^24^ Yet sharing those notes could often save time for other researchers and make the scientific endeavor more transparent, traceable, and tractable. Just as the publication of negative results could help alleviate the problem of publication bias,^21,44–46^ the sharing of critical reading notes (thus being exposed to the experience and reactions others had when reading those articles) have the potential to improve methodologies and scientific practices.^47^ Sharing all results, discussing them, even after publication, lays the foundation for a research environment that is not only more open but also more genuinely scientific (in the sense of fostering that Merton-like organized skepticism mindset). In light of these considerations, our hypothesis is that if more nano-scientists engage in post-publication peer review, the usability and quality of research can improve.

A practical approach is to use PubPeer.com, a website that allows discussion of any scientific article as long as it has a DOI, a PubMed ID or an ArXiv ID. The PubPeer site, which effectively provides an instance of post-publication peer review, originated from an idea of neuroscientist Brandon Stell (aided by Richard and George Smith and later by colleagues such as Boris Barbour, and legal expert Gabor Brasnjo). It is a California-registered public-benefit corporation with 501(c)(3) nonprofit status in the United States.^48^ PubPeer offers an online platform where comments are posted, authors are notified that their work has been commented on, and a discussion space can be created that can lead to clarification of unclear points, up to correction of the article (if errors are revealed and authors, along with editors take the responsibility to make a correction). In addition, PubPeer extensions^49^ for web browsers allow readers of any scientific article to be automatically notified if the publication they are reading (or the articles cited) has comments on PubPeer.

## The NanoBubbles’ post-publication peer review initiative

We, authors of this paper, are members of the NanoBubbles project, a European Research Council Synergy project that focuses on how, when, and why science fails to correct itself. The project is highly interdisciplinary and, in particular, extends to the human and social sciences but stems from the experience of a nano scientist (RL) struggling to challenge errors and misconceptions in research.^50,51^ NanoBubbles includes a post-publication peer review and a replication initiative (amongst others sub-projects). Both aim to help nano scientists, directly and through changes in practices, navigate a field where information bubbles, hype, errors, and misconceptions are common. To demonstrate the benefit of contributing to PubPeer, to-date, we have decided to focus on one specific scientific question of relevance to many proposed applications of nanoparticles: their access to the interior of the cells. We consider, in particular, articles that report nanoparticles-based sensing of cytosolic analytes (mRNAs, ions, pH). In spite of the fact that efficiently delivering nanoparticles to the cytosol is, at best, highly challenging, there are over a thousand articles that report intracellular sensing with nanoparticles. We critically read some of those articles and share our comments on PubPeer, after which authors are notified and have the opportunity to respond. Other scientists can also join in the discussion (anonymously or not). Thus, on this particular disputed nanoscience topic, we are building a common resource (42 comments so far), which we hope would be useful to any scientist in this field or preparing to enter this field. It could also be of interest as a teaching resource for training young researchers.

To maximize transparency and emphasize the scholarly nature of our comments, we sign our reviews and include conflict of interest declarations and author's contributions statements. We start our review with a summary of the article and its main claims to help the reader gain a clear understanding of the study's objectives and findings. We then report our critical observations. Often the response of the scientific community to general concerns about the need for better practices, specifically in nanoscience, has been to insist on higher standards of characterization, *e.g.*, with the introduction of minimum reporting guidelines.^52,53^ Whilst our comments do address critical aspects related to materials’ synthesis and characterization, they also consider other fundamental issues. These include basic conceptual problems, which are sometimes ignored or minimized, as well as the justification for the study: why is this sensor needed? Is its complexity justified by something else than the need for novelty? How does it compare with other methods currently available? Whilst a detailed analysis of the various points discussed in our comments is beyond the scope of this article, Table 2 highlights some of the issues encountered.

**Table 2:** Common issues described in NanoBubbles post-publication peer review comments

Description of the issue	Exemplary NanoBubbles comments posted in PubPeer
**Access to the cytosol**	
*Mechanism of uptake*: nanoparticles usually enter cells by endocytosis thus ending up sequestered in endosomes. However, many articles do not discuss the mechanism of uptake, giving the impression of passive crossing.	Interactions of neutral gold nanoparticles with DPPC and POPC lipid bilayers: simulation and experiment (https://pubpeer.com/publications/7BC6FBD99F859291D5062CAD6F0D1D)
*Endosomal escape*: the intracellular detection of molecules, ions and pH monitoring in the cytosol is only possible if the nanoparticles escape the endosomes. Yet many articles do not mention endosomal escape, whilst others claim access of the nanoparticles based on weak evidence.	Hybrid nanoparticle pyramids for intracellular dual MicroRNAs Biosensing and bioimaging (https://pubpeer.com/publications/086A980BF38D5ECF529C8FF4EC91D4) Graphene oxide-peptide conjugate as an intracellular protease sensor for caspase-3 activation imaging in live cells (https://pubpeer.com/publications/96168F895CFB10A6828FD347B87376)

**Rationale**	
What is the justification for the work? If the aim is to make a new sensor, there should be an analysis of the need, and a comparison with existing alternatives.	FRET nanoflares for intracellular mRNA detection: avoiding false positive signals and minimizing effects of system fluctuations (https://pubpeer.com/publications/531E227CABF29AFB8CC987DE66AA02)

**Methodology**	
The experimental set-up is not clear, important protocol information is missing. These issues make it difficult to replicate the experiments.	Promoted “Click” SERS detection for precise intracellular imaging of caspase-3 (https://pubpeer.com/publications/D0F6E93F8B0CA6AED5A9C780BE8EEE)

**Characterization**	
The samples are not properly characterized, there is a lack of standard measurements, or the data were not clearly discussed.	Aptazyme–gold nanoparticle sensor for amplified molecular probing in living cells (https://pubpeer.com/publications/1871F5D19263598D608CD9FD618ACE)

**References**	
There is no relation between the reference and the corresponding sentence in the article, or there are missing references. Important claims are made referring to “it is discussed” or “previous work”, but no reference. Some established methods or knowledge are mentioned, but the original work is not cited.	A multicolor nanoprobe for detection and imaging of tumor-related mRNAs in living cells (https://pubpeer.com/publications/6E38EC6645D900AC45B85CB321FE46)

To date, limited but encouraging engagement has taken place with authors responding to our comments. In one case, the authors decided to issue a correction which has been published (Park *et al.*, 2021)^54^ whilst in another, authors have announced that they would contact the editorial office to request a correction (comment on Wei *et al.*, 2020).^55^ Beyond authors, other scientists added further comments to our post-publication peer reviews.

## Perspective

In this commentary, we argued that scientific self-correction mechanisms need to be improved. Among the practices that can be pursued by any individual researcher, post-publication peer-review, such as that on PubPeer, provides a valid, and above all accessible to anyone, strategy to achieve this goal. Whilst this approach is unlikely to be the method that solves the problem of uncorrected science (since it is a complex issue and touches on other aspects that we have not discussed here), we hope that this article will increase attention to these issues and inspire more researchers in the nanoscience community to engage and participate in this process. Thus, we invite readers to share their thoughts on articles that concern nanoparticle-based intracellular sensing or any other topics of their interest, commenting on methodological issues, but also on other aspects, including the rationale of the research. In our opinion, such commitment could help to move the scientific community forward, both in terms of shared understanding and common research standards.

## Conflicts of interest

There are no conflicts to declare.
